# The Mechanical Properties of Thin-Walled Specimens Printed from a Bronze-Filled PLA-Based Composite Filament Using Fused Deposition Modelling

**DOI:** 10.3390/ma16083241

**Published:** 2023-04-20

**Authors:** Jerzy Bochnia, Tomasz Kozior, Malgorzata Blasiak

**Affiliations:** Faculty of Mechatronics and Mechanical Engineering, Kielce University of Technology, 25-314 Kielce, Poland; jbochnia@tu.kielce.pl (J.B.); mblasiak@tu.kielce.pl (M.B.)

**Keywords:** three-dimensional printing, FDM, PLA + bronze, polymers

## Abstract

This article focuses on the mechanical property analysis of important models omitted in many scientific papers (thin-walled specimens) printed from innovative material—such as PLA + bronze composite—using fused deposition modelling technology. It discusses the printing process, the measurement of the specimen geometry, the static tensile strength tests and the microscopic examinations conducted with a scanning electron microscope. The findings of this study could be used as an input to further research into the accuracy of filament deposition and the modification of base materials with bronze powder and for the optimization of the machine design, e.g., with the use of cell structures. The experimental results indicated that the thin-walled models fabricated using FDM showed substantial differences in tensile strength, depending on the specimen’s thickness and the printing orientation. It was shown that it was not possible to test thin-walled models located on the building platform along the *Z* axis due to the lack of sufficient adhesion between the layers.

## 1. Introduction

Rapid technological developments observed in the area of additive manufacturing (AM) have considerably increased its potential and popularity. Additive manufacturing is particularly suitable for use instead of other time-consuming, complicated and expensive operations. FDM (fused deposition modelling) and SLS (selective laser sintering) are the two most popular additive manufacturing technologies. FDM is commonly used in industrial applications because it allows for elements to be subjected to further processes, such as unconventional chemical machining and conventional machining. FDM is frequently used to rapidly produce one-of-a-kind items with no geometric limitations and with no need to mechanically finish the surface or drill holes. Elements fabricated in this way are characterized by their high strength. The advantages of FDM include the availability of a wide variety of raw materials and a relatively low cost of fabrication. The technology offers the possibility to print prototypes of mechanisms in order to check their functionality. This method is also well-suited for selecting the best internal structures for products, e.g., a honeycomb core, which not only reinforces an element, but also reduces its weight; this fact is of great significance in the case of tools used by humans for production or assembly purposes [1].

Since the printing equipment is relatively cheap and easy to operate, and there is a wide range of raw materials available for use, FDM is stated to be a suitable method to print thermoplastics. Three-dimensional-printed polymer-based elements have been reported to have relatively good surface qualities and geometrical and dimensional accuracy. Thermoplastic polymers, which are available in different types and forms, can be used to build objects composed of simple or intricate shapes, making them extremely common in the manufacturing sector. Solid models composed of powder materials can take the form of thin-walled elements, which can be fabricated in series. New, advanced materials are being introduced to improve the durability, reliability and functionality of printed elements.

Thermoplastics and their composites are becoming increasingly popular in manufacturing applications, e.g., for sleeves or bearings; they are also becoming more and more common in the construction and automotive sectors, with examples of the latter including dashboards, door panels, roof linings and other car exterior and interior parts [2]. There are different methods of manufacturing models from composite materials. In the case of 3D printing technology and the FDM method, it is possible to extrude material from a previously prepared filament that is a composite material using a single-material delivery system; it is also possible to deliver the base material and component through separate systems, and then mix them in an extruder. In the presented article, it was decided to use the first variant with a composite filament for printing the samples.

PLA is suitable for 3D printer filaments mainly because of its relatively good mechanical properties and biodegradability. Its tribological properties, however, have not been thoroughly tested yet [3]. PLA is a semicrystalline thermoplastic polyester. It is also a biopolymer, which is easy to create using natural materials, such as starch and sugar, and that can be decomposed through composting under industrial conditions [4].

PLA-based filaments are relatively strong; however, because of their low elasticity, hole drilling in elements composed of this polymer is not recommended as they may melt, crumble or break. In some applications, the high stiffness of models printed from a PLA filament is vital, because they do not deform under normal conditions [5].

The mechanical properties of PLA-based elements may be different in different directions (material anisotropy), which may have an effect on their quality [6,7]. The properties of thermoplastic polymers can be improved by adding some fibres or increasing the infill percentage.

Fibre- or nanoreinforced composites are characterized by their higher efficiency and functionality [8]. Some of the research in this area has aimed to determine the influence of different types of reinforcements, e.g., fibre glass [9], carbon fibre [10,11], wood [12] or microfibers. As reported in [13], PLA reinforced with graphene showed a 14% higher resistance to wear and a 65% lower coefficient of friction, which resulted in the composite being suitable for orthopaedic applications. From the literature on the subject, it is clear that the use of bronze may considerably improve the tribological properties of polymer-based composites. The presence of bronze contributes to the better sliding behaviour of the material, which is a consequence of the lower metal-to-metal friction. The experiments described in [14] revealed that the tribological properties of the PLA + bronze composite were largely dependent on the content of bronze and the printing orientation. The tests showed that the presence of bronze particles acting as the reinforcement for PLA was responsible for the much lower depth of wear, which suggested an improvement in the tribological properties of the material. Apart from the presence of bronze, the printing orientation and the indirectly layered structure of the sliding contact surface had a significant influence on the coefficient of friction, the wear resistance and other mechanical properties. The findings suggest that the printing direction should be optimized to allow the product to be suitable for a specific application. Another conclusion drawn from that research is that 3D-printed components could potentially be used for sliding applications in industry, e.g., for bearings and sleeves operating at low speeds.

As indicated in [15], the adhesive wear of bearings composed of bronze-reinforced polymers was lower because of the higher resistance of the bronze to wear. The study described in [16] revealed that the highest resistance to wear was observed for PTFE reinforced with 60% bronze. Under lower loading conditions (less than 30 N), the coefficient of friction decreased. When the loading was higher, i.e., greater than 30 N, the coefficient of friction remained stable. The loading had a greater effect on the wear than the sliding speed.

The mechanical properties of a PLA + bronze composite subjected to compressive loading were also analysed in [17]. The mechanical properties of specimens printed from bronze-filled PLA were compared with those of specimens created from sintered bronze powder (CuSn10). It was found that a higher infill percentage and a lower layer thickness contributed to 17% higher tensile stress. The tests described in [18,19] aimed to determine the mechanical properties of elements 3D printed from a PLA filament reinforced with 14% bronze. The studies dealt with the influence of the nozzle’s temperature and the layer thickness on the material’s behaviour under bending and compressive loading conditions. From the observations, it was apparent that the nozzle temperature highly affected the mechanical properties of the 3D-printed objects. The layer thickness and the printing direction were also responsible for the tensile strength of the prints. Hole drilling in additively manufactured elements may also contribute to material delamination, as described in [20]. The delamination percentage was found to be dependent mainly on the feed, the cutting speed and the hole diameter.

The aim of the research discussed in [21] was to determine the relationships between the variable printing/sintering directions and the properties of the sintered metal specimens. The printing was performed using fused filament fabrication (FFF) from a metal bronze/PLA hybrid filament. It was found that the printing/sintering orientations could influence the mechanical properties and porosity of the metallic elements.

Extensive research is required to determine the properties of fibre-reinforced composite elements fabricated through 3D printing. The mechanical properties, including tensile strength, compressive strength, flexural strength, fatigue strength and impact resistance, are of particular importance [22]. Depending on the function that the element performs, various quality criteria are adopted regarding the dimensional and shape accuracy, mechanical properties, etc. The quality of additively manufactured objects is largely dependent on their geometric, dimensional accuracy and surface texture (e.g., surface roughness and waviness), as indicated in [23,24]. From a review of the literature, it was evident that by modifying the printing parameters, for example, reducing the layer thickness, increasing the nozzle diameter, controlling the infill percentage and selecting the right printing orientation and infill pattern, it is possible to considerably improve the tensile strength of printed elements and reduce their porosity.

This article considers the mechanical properties (tensile tests) of 3D-printed thin-walled elements fabricated using FDM from a PLA + bronze filament. This material was selected for testing due to its potentially large number of applications in the construction of thin-walled models that require to be resistant to abrasion, as indicated by the addition of bronze and the review of the literature. In addition, this material is a composite, so the evaluation of the anisotropy of the mechanical properties depending on the printing direction was analysed, which is a key scientific problem related to FDM/FFF technology. Thin-walled structures in the era of the optimization of LEAN manufacturing are an important component of manufactured products for applications in, for example, medicine, the aviation industry and the automotive industry.

A novelty in the presented work is the analysis of the PLA + bronze composite material, taking into account the possibility of building thin-walled elements (with a thickness of 1, 1.4 and 1.8 mm) in three directions and the analysis of the structure using scanning microscopy. The analysis of new materials very often omits the assessment of the influence of the printing direction, which, as shown through the test results presented in this article, has a very large impact on the properties of the models produced. Thanks to the unification of the research methodology for thin-walled models, presented in this article, it is possible to compare the results for other materials in terms of the construction of thin-walled models and cellular structures.

## 2. Materials and Methods

The design of the experimentation involved designing CAD specimens, the appropriate saving of STL files, the production of models using 3D printing technology with selected technological parameters and conducting research, a statistical analysis and conclusions.

**Figure 1:**
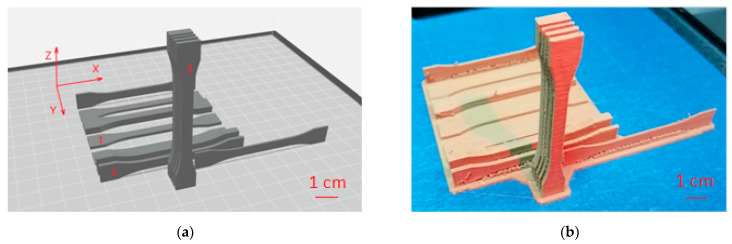
Specimens on the virtual build platform of the fifth-generation MakerBot Replicator. (**a**) Arrangement of models in the MakerBot software printed along: 1—the *X* axis; 2—the *Y* axis; and 3—the *Z* axis; (**b**) a series of 3D-printed specimens.

The specimens under analysis were prepared and measured in compliance with the ISO 527 standard [25], specifying the requirements for the determination of the tensile strength of polymer-based materials. The specimens were created from a commercially available filament (MetalFil Ancient Bronze, produced by Formfutura in Nijmegen, The Netherlands). The material in the form of a filament with a diameter of 1.75 mm is a commonly used PLA with 80% bronze. The specimens were oriented in three directions on the build platform of the 3D printer. There were five specimens of each variant with a total of fifteen specimens. The printing was carried out using a fifth-generation MakerBot Replicator printing machine (Brooklyn, NY, USA). The arrangement of the models on the build platform of the 3D printer is visualized in Figure 1.

The specimens composed of MetalFil (bronze-filled PLA) were fabricated using standard process parameters, as recommended by the material producer [26]: layer thickness—0.2 mm; nozzle temperature—220 °C; and nozzle diameter—0.4 mm. Some of the properties of the material tested are provided in Table 1.

The macrostructure of the fractured specimens was analysed using a stereoscopic microscope. More detailed observations of the fracture area were performed with a JEOL JSM-7100F scanning electron microscope (SEM) to determine the characteristic features of the material macrostructure in the cross-sections.

## 3. Results

### 3.1. Thickness and Width Measurements

The thickness and width measurements were performed for each specimen at three points within the gauge length. The measurements were conducted using a micrometre with a reading accuracy of 0.01 mm. The data were used to calculate the mean thickness, *ā*, and the mean width, $\overset{-}{b}$. The information provided in Table 2 contains the specimen number in a series and the printing direction (column no.), the nominal thickness (column *ā*) and the nominal width (column $\overset{-}{b}$). For example, the specimen marked 1.4 3X represents the specimen with a nominal thickness of 1.4 mm printed in the X direction as the third in a series. The nominal thickness and the other nominal dimensions were the values saved in a CAD file converted to an .stl file to be read using the printer software. The actual dimensions, i.e., the dimensions of the as-printed specimens, were slightly different from the nominal values, which was due to the limitations of the 3D printer used. The same problem could be observed for all 3D printers.

Table 2 provides the dimensions of only one specimen printed in the Z direction, i.e., that which fractured correctly during the static tensile strength tests. The other specimens printed in the Z orientation prepared for the tests were measured to determine their dimensions, but they were so brittle that they cracked or fractured while being mounted in the tensile grips of the universal testing machine. For this reason, their dimensions were not included in Table 1.

### 3.2. Tensile Test

The mechanical property test included a static tensile strength analysis. The main parameter was a speed equal to 1 mm/min, according to the ISO standard for specimens shaped in the XY direction. For the test, we used the Ispekt mini 3 kN testing machine produced by Hegewald & Peschke GmbH. According to Formula (1), the tensile strength (*R_m_*) was determined using the software LabMaster:

(1) $$R_{m}=\frac{F_{m}}{\bar{a}\bar{b}},$$

where *F_m_*—maximum force; *ā*—mean measured thickness of a specimen; $\overset{-}{b}$—mean measured width of a specimen.

The mean values of the specimens’ dimensions (width and thickness) are presented in Table 1. Then, the data were exported to the analysis software to calculate the tensile strength, *R_m_*, and draw the stress–strain curve. In this type of test, the same nominal dimensions are generally set for a whole series of specimens. The dimensional errors need to be taken into account in the calculations. In the case of the thin-walled specimens, however, this approach could have affected the calculation results.

The modulus of elasticity, *E*, of each specimen was calculated using the regression analysis for the straight-line portion of the respective stress–strain curve.

**Figure 2:**
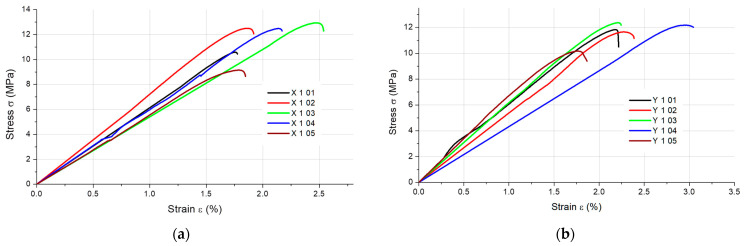
Stress–strain curves for the 1 mm thick specimens composed of PLA + bronze printed in (**a**) the X orientation and (**b**) the Y orientation.

**Figure 3:**
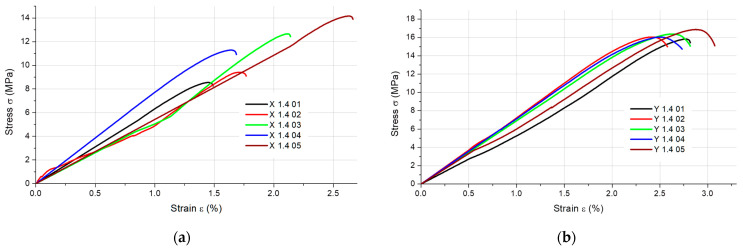
Stress–strain curves for the 1.4 mm thick specimens composed of PLA + bronze printed in (**a**) the X orientation and (**b**) the Y orientation.

**Figure 4:**
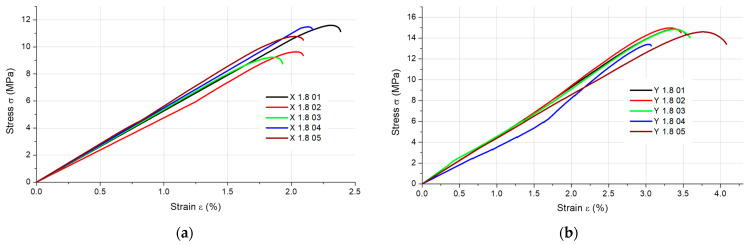
Stress–strain curves for the 1.8 mm thick specimens composed of PLA + bronze printed in (**a**) the X orientation and (**b**) the Y orientation.

**Figure 5:**
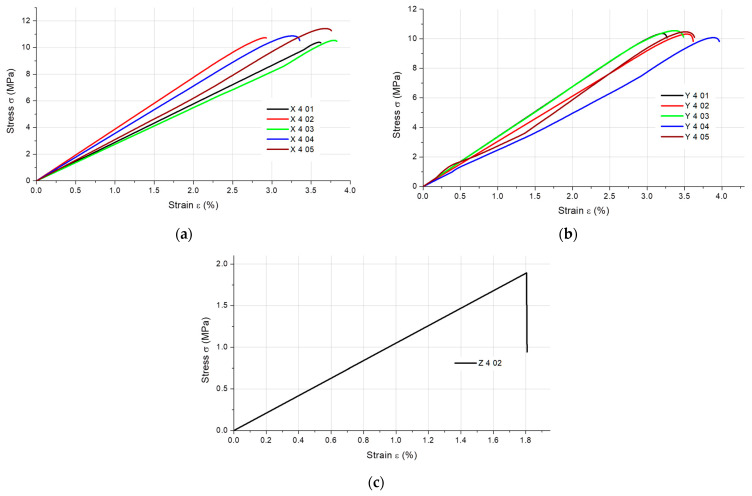
Stress–strain curves for the 4 mm thick specimens composed of PLA + bronze printed in (**a**) the X orientation, (**b**) the Y orientation and (**c**) the Z orientation (only one specimen tested).

The results of the static tensile strength tests obtained for the PLA + bronze composite specimens are illustrated in Figure 2, Figure 3, Figure 4 and Figure 5, according to each specimen’s thickness and printing orientation.

The ultimate tensile strength, *R_m_*, and the maximum elongation at failure in %, *ε_m_*, which occurred under the maximum tensile loading, reported for the PLA + bronze composite specimens, are shown in Table 3.

Table 4 shows the values of the modulus of elasticity, *E,* estimated using the regression analysis on the basis of the static tensile test results obtained for the different PLA + bronze specimens.

Table 3 and Table 4 show the tensile test results obtained for a specimen printed in the Z direction. As mentioned above, there was only one specimen tested successfully. Since the tensile strength was minimal, microscopic examinations were conducted to explain the reason for that, as well as the fragility of the specimens printed in the Z orientation.

### 3.3. Microscopy

**Figure 6:**
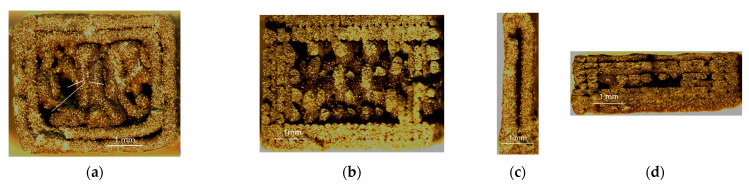
Cross-sections of the PLA + bronze specimens after fracture observed by means of a stereoscopic microscope, magnification ×12; (**a**) specimen 4.0 2Z, with visible black areas indicating lack of filament (1); (**b**) specimen 4.0 5Y; (**c**) specimen 1.0 2Z; (**d**) specimen 1.8 5X.

The images in Figure 6 depict the fracture area examined with a stereoscopic microscope.

Figure 6a,b show the cross-sections of the 4 mm thick specimens printed in the Z and Y orientations, respectively. As can be seen from Figure 6a, there were some gaps between the layers and lack of an infill (marked 1). The lack of an infill and gaps between the filament layers were also visible in the 1 mm and 1.8 mm thick specimens (Figure 6c,d, respectively), which suggests that they were independent of the printing direction.

**Figure 7:**
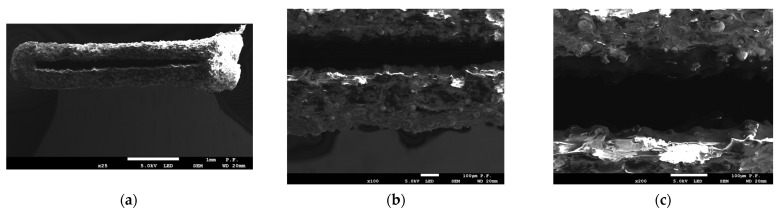
Cross-section of specimen 1.0 2Z composed of PLA + bronze after fracture observed with a scanning electron microscope. Observation direction—perpendicular to the cross-section; (**a**) magnification ×25; (**b**) magnification ×100; (**c**) magnification ×200.

**Figure 8:**
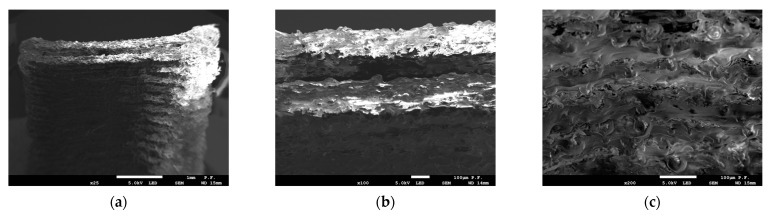
Cross-section of specimen 1.0 2Z composed of PLA + bronze after fracture observed with a scanning electron microscope. Observation direction—at an angle of 45° to the cross-section; (**a**) magnification ×25; (**b**) magnification ×100; (**c**) magnification ×200.

**Figure 9:**
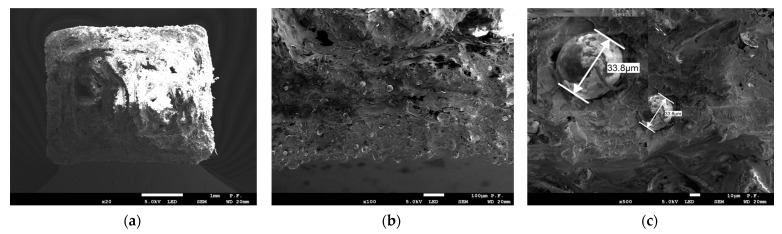
Cross-section of specimen 4.0 2Z composed of PLA + bronze after fracture observed with a scanning electron microscope. Observation direction—perpendicular to the cross-section; (**a**) magnification ×25; (**b**) magnification ×100; (**c**) magnification ×500.

**Figure 10:**
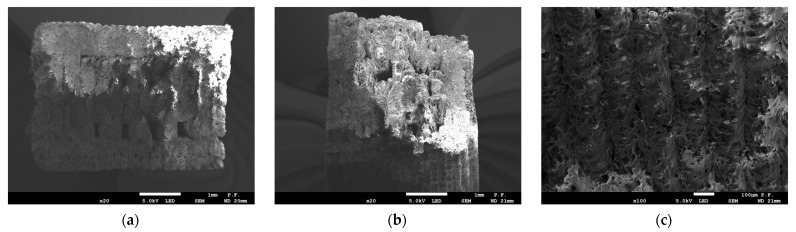
Cross-section of specimen 4.0 2Y composed of PLA + bronze after fracture observed with a scanning electron microscope; (**a**) observation direction—perpendicular to the cross-section; magnification ×25; (**b**) observation direction—at an angle of 45° to the cross-section; magnification ×25; (**c**) observation direction—at an angle of 45° to the cross-section; magnification ×100.

**Figure 11:**
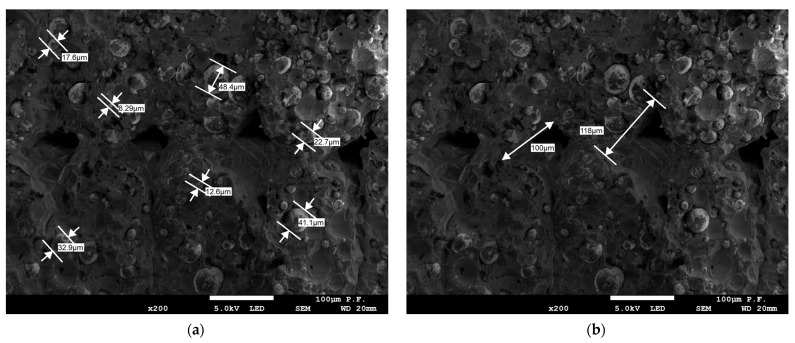

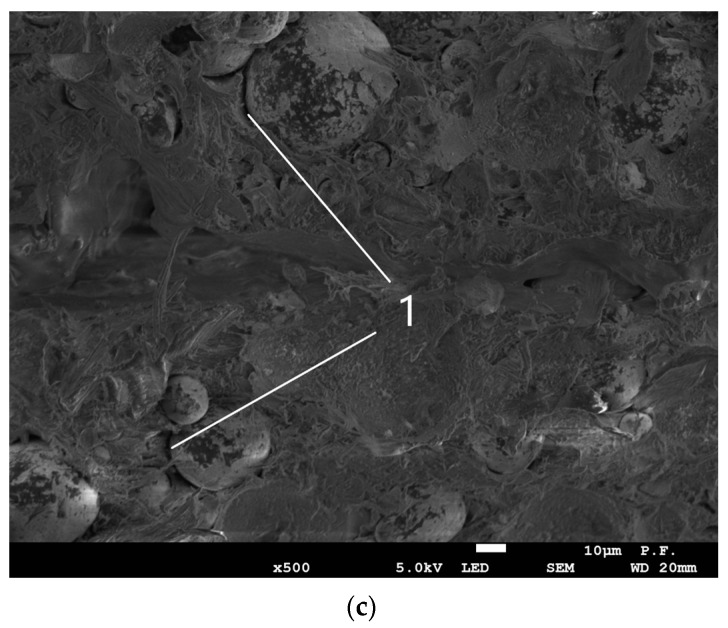
Cross-section of specimen 4.0 2Y composed of PLA + bronze after fracture analysed with a scanning electron microscope; (**a**) observation direction—perpendicular to the cross-section; diameters of the bronze powder grains; magnification ×100; (**b**) observation direction—perpendicular to the cross-section; gap sizes; magnification ×100; (**c**) observation direction—at an angle of 45° to the cross-section; magnification ×100.

The images of fractured specimens in Figure 7, Figure 8, Figure 9, Figure 10 and Figure 11 were obtained with a scanning electron microscope.

Figure 7 and Figure 8 show the cross-sections of the fractured 1 mm thick specimens printed in the Z direction observed at 90° and 45°. Layer separation was clearly visible, and the gaps between the filament layers, which occurred in all the specimens, irrespective of the printing direction, were responsible for the lower tensile strength. As can be seen from Figure 8a, the defects occurred at the contact area between the layers of filament.

Figure 10 and Figure 11 show the cross-sections of the reference 4 mm thick specimens. The sizes of the bronze powder grains and gaps between the filament layers were measured, as illustrated in Figure 11a,b, respectively. The gaps between the powder grains and PLA visible in Figure 11c indicated that there was no adhesive bonding between them. The grain size of the bronze powder ranged from 8.29 µm to 48.8 µm (Figure 11a). The gaps between the filament layers were different, for instance, 100 µm and 118 µm (Figure 11b). Thus, it could be concluded that the poor tensile strength of the specimens tested was a result of the poor adhesion of the powder reinforcement to the base material, the high fibre volume ratio, i.e., the high percentage of bronze volume in the entire volume of the PLA-based composite material, and the occurrence of numerous gaps between the filament layers.

## 4. Discussion

Experimental studies of 3D-printed thin-walled elements, for instance, those described in [9,11], focus on two main areas: the geometrical and dimensional accuracy and the mechanical properties. This is mainly because of the specific nature of the additive manufacturing process. Three-dimensional printing involves extruding a filament layer by layer onto a build platform to create geometrically and dimensionally accurate elements and fibres bonded adhesively and/or cohesively. Three-dimensional-printed elements have a specific macrostructure, which is why it is important to study their mechanical properties.

The relative percentage changes in the thickness and width, Δ*a* and Δ*b*, respectively, were determined to assess the measurement data for each measurement series. The following formulas were used [9]:

(2) $$\Delta a_{X,Y,Z}=\frac{\left|a-{\bar{a}}_{X,Y,Z}\right|}{a}\cdot 100\%,$$

where *a*—nominal thickness of the specimen, e.g., *a* = 1.4 mm; ${\bar{a}}_{X,Y,Z}$—mean thickness in a given measurement series on the basis of Table 1 or Table 2. For example, for a specimen printed in the X orientation with *a* = 1.4 mm (Table 1), ${\bar{a}}_{X}$ = 1.59 mm,

(3) $$\Delta b_{X,Y,Z}=\frac{\left|b-{\bar{b}}_{X,Y,Z}\right|}{b}\cdot 100\%,$$

where *b*—nominal width of the specimen, e.g., *b* = 5 mm; ${\bar{b}}_{X,Y,Z}$—mean width of a specimen in a given measurement series on the basis of Table 1 or Table 2. For example, for a specimen printed in the X orientation with b = 5 mm and *a* = 1.4 mm (Table 1), ${\bar{b}}_{X}$ = 5.37 mm.

**Figure 12:**
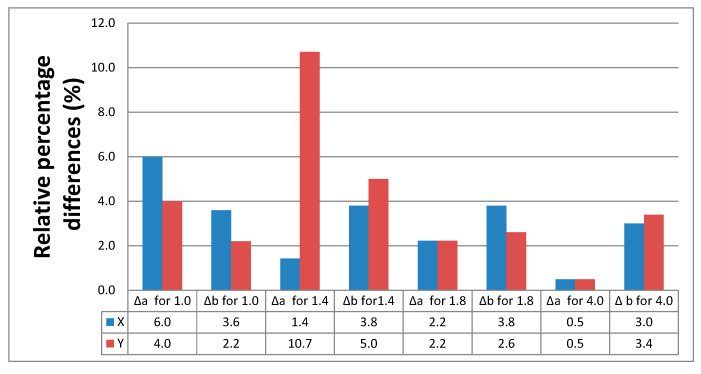
Relative percentage differences in thickness and width of the PLA + bronze specimens.

The relative percentage differences in the thickness (Δ*a*) and width (Δ*b*) calculated for all the measurement series are illustrated in Figure 12. The values were based on the data shown in Table 2.

The smallest relative percentage differences in thickness were observed for the specimens with a nominal thickness of 4 mm printed in the X or Y directions. A very large difference was reported for the specimens 1.4 mm in thickness printed in the Y direction. Generally, larger differences between the nominal and actual dimensions were observed for the thin-walled specimens.

**Figure 13:**
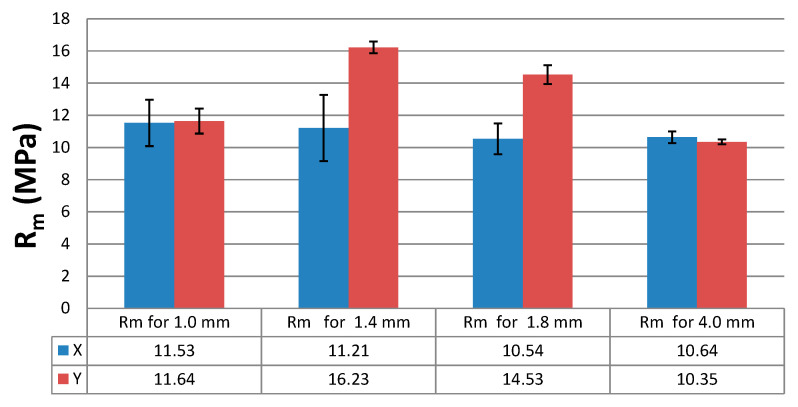
Tensile strength, Rm, of the PLA + bronze specimens, according to their thickness and the printing direction.

From the analysis of all the specimen series, it was clear that the thin-walled specimens with a thickness of 1.4 mm or 1.8 mm differed considerably in tensile strength, and the values depended on the printing direction. The results of the tensile strength tests were compared in Table 3 and in the bar chart in Figure 13. It should be mentioned that no specimen printed in the Z direction was analysed because they all cracked or broke while being mounted in the tensile grips of the universal testing machine. The tensile test was performed only for one specimen (Figure 5c). Its strength was reported to be minimal, which was due to the model building method, the printing orientation (along the *Z* axis) and the layer-by-layer arrangement of the filament. If a composite material contains more powder filling, as was the case with PLA + 80% bronze, the weak adhesive bonds become even weaker. As can be seen from the microscopic images in Figure 9c and Figure 11c, there were gaps between the base material, PLA, and the powder grains, indicating no or poor adhesive bonding.

**Figure 14:**
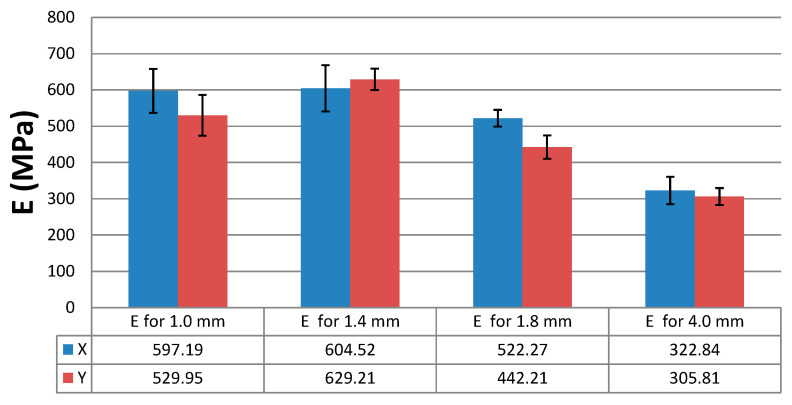
Modulus of elasticity, E, of the PLA + bronze specimens, depending on their thickness and the printing orientation.

Based on the tests carried out for the PLA + bronze material and the results presented in Figure 14, we conclude that the modulus of elasticity, E, was reported to be dependent on the printing direction and the specimen’s thickness. The differences reached several percent (up to 18%). Interestingly, the values were lower for the thickest PLA + bronze specimens, i.e., those 4 mm in thickness, than for the thin-walled models, as shown in Figure 14. Generally, the modulus of elasticity was lower for the specimens printed in the Y orientation than for those fabricated in the X orientation (Figure 14), which was mainly due to the arrangement of the filaments. In the case of specimens with a thickness of 1.4 mm, the modulus of elasticity was slightly higher in the Y direction than in the X orientation.

The highest modulus of elasticity was obtained for the specimens with a thickness of 1.4 mm. The differences in the mechanical properties of the thin-walled specimens resulted mainly from the filament arrangement strategy and, consequently, the presence of gaps between the layers, visible, for example, in the microscopic images in Figure 6 and Figure 7. The difference in the lower Young’s modulus for the 4 mm thick specimens (Figure 14) was due to the way the filament layers were distributed during printing, as observed. The filament for the thin-walled specimens was placed along the length of the specimens, and for the specimens with a thickness of 4 mm (solid), the model was divided into a shell and a filling (see Figure 6).

**Figure 15:**
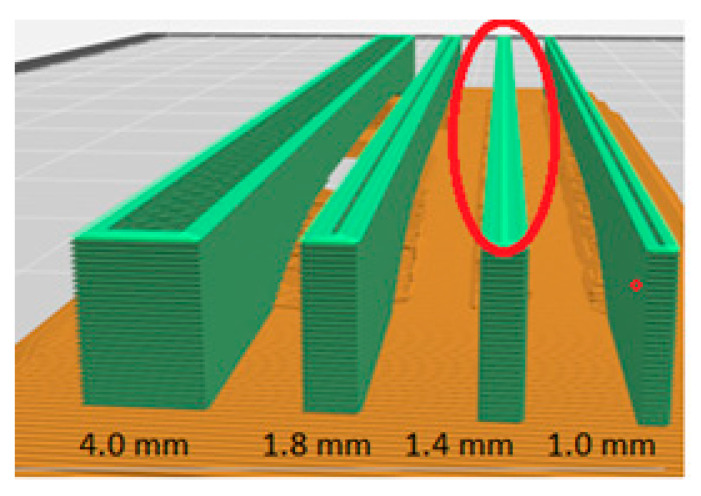
Simulation of 3D printing of specimens in the Y orientation.

Below, in Figure 15, a view of the printout simulation of the specimens placed on the platform in the *Y* axis is presented.

Figure 15 shows simulations of printing specimens in the *Y* axis for all analysed thicknesses. On the cross-section of the built layers for a specimen with a thickness of 1.4 mm, it can be seen that the program introduced additional overlapping (marked in red) layers inside the model (in the infill). For other types of specimens, there was no overlapping of the layers within the model, and the paths were ordered and formed into a solid. In relation to specimen 1.4, we were dealing with an increase in the “a” dimension in relation to the CAD model due to the additional overlapping of model layers in this direction, which is shown in Figure 14 in the form of dimensional errors.

## 5. Conclusions

The study of the mechanical properties of thin-walled specimens fabricated from a bronze-filled PLA-based composite filament using FDM confirmed that this innovative engineering material with orthotropic properties requires thorough examination. Several conclusions could be drawn from the research results.

Firstly, the high content of bronze powder used as the filling was responsible for a considerable decrease in the tensile strength of the specimens printed in the Z direction; this factor affected the strength of both the thin-walled specimens and those 4 mm in thickness, used for reference purposes. The analysis of the tests of specimens manufactured in three directions indicated a very high anisotropy, especially in the Z printing direction, which prevented both the process of cleaning the support material and further tests; this is the least recommended positioning for models on a building platform.

Secondly, the poor adhesion of the powder to the base material and the presence of numerous gaps, observed in microscopic images, contributed to the lower tensile strength of the composite. The gaps between the filament layers were caused due to the specific motion of the print head controlled with a special algorithm. The tiny gaps had less effect on the mechanical properties of the thicker elements. In the case of the thin-walled prints, however, their influence was substantial.

It seems that for the thin-walled models, software modifications or an additional nodule, for example “thin walls”, should be introduced by the 3D printer producer, allowing to avoid this unfavourable phenomenon, which, in the future, is planned for further research and implementation by the research team.

## Figures and Tables

**Table 1:** Selected properties of the Formfutura MetalFil Ancient Bronze filament.

Properties	Value	Standard
Specific gravity	3.5 g/cc	ISO 1183 [27]
Tensile strength	19.0 MPa	ISO 527 [25]
Tensile modulus	3990 MPa	ISO 527 [25]
Elongation at break	8%	ISO 527 [25]
Viscat softening temp	±65 °C	ISO 306 [28]

**Table 2:** Dimensions of the PLA + bronze specimens prior to tensile testing.

No.·	*ā* (mm)	$\bar{b}$ (mm)	No.·	*ā* (mm)	$\bar{b}$ (mm)	No.·	*ā* (mm)	$\bar{b}$ (mm)	No.·	*ā* (mm)	$\bar{b}$ (mm)
1.0 1X	1.09	5.24	1.4 1X	1.43	5.23	1.8 1X	1.78	5.19	4.0 1X	4.00	5.23
1.0 2X	1.07	5.28	1.4 2X	1.39	5.18	1.8 2X	1.85	5.19	4.0 2X	3.97	5.11
1.0 3X	1.06	5.12	1.4 3X	1.46	5.15	1.8 3X	1.87	5.19	4.0 3X	4.05	5.13
1.0 4X	1.04	5.16	1.4 4X	1.39	5.25	1.8 4X	1.81	5.22	4.0 4X	4.04	5.13
1.0 5X	1.06	5.11	1.4 5X	1.43	5.14	1.8 5X	1.87	5.17	4.0 5X	4.03	5.14
$\bar{x}$	1.06	5.18	$\bar{x}$	1.42	5.19	$\bar{x}$	1.84	5.19	$\bar{x}$	4.02	5.15
SD	0.016	0.067	SD	0.027	0.043	SD	0.036	0.016	SD	0.029	0.042
1.0 1Y	0.97	5.08	1.4 1Y	1.56	5.27	1.8 1Y	1.88	5.13	4.0 1Y	3.99	5.15
1.0 2Y	0.98	5.09	1.4 2Y	1.54	5.27	1.8 2Y	1.83	5.13	4.0 2Y	4.03	5.15
1.0 3Y	0.94	5.13	1.4 3Y	1.55	5.22	1.8 3Y	1.83	5.13	4.0 3Y	4.02	5.18
1.0 4Y	0.98	5.13	1.4 4Y	1.55	5.24	1.8 4Y	1.82	5.14	4.0 4Y	4.02	5.16
1.0 5Y	0.95	5.14	1.4 5Y	1.54	5.18	1.8 5Y	1.83	5.13	4.0 5Y	4.02	5.19
$\bar{x}$	0.96	5.11	$\bar{x}$	1.55	5.25	$\bar{x}$	1.84	5.13	$\bar{x}$	4.02	5.17
SD	0.016	0.024	SD	0.007	0.034	SD	0.021	0.004	SD	0.014	0.016
1.0 2Z	-	-	1.4 2Z	-	-	1.8 2Z	-	-	4.0 2Z	4.04	5.30

**Table 3:** Ultimate tensile strength and the maximum elongation at failure in % for the PLA + bronze specimens.

No.·	*R_m_* (MPa)	*ε_m_* (%)	No.·	*R_m_* (MPa)	*ε_m_* (%)	No.·	*R_m_* (MPa)	*ε_m_* (%)	No.·	*R_m_* (MPa)	*ε_m_* (%)
1.0 1X	10.59	1.8	1.4 1X	8.55	1.4	1.8 1X	11.60	2.3	4.0 1X	10.40	3.6
1.0 2X	12.50	1.9	1.4 2X	9.41	1.7	1.8 2X	9.64	2.0	4.0 2X	10.74	2.9
1.0 3X	12.93	2.5	1.4 3X	12.66	2.1	1.8 3X	9.23	1.9	4.0 3X	10.54	3.8
1.0 4X	12.48	2.1	1.4 4X	11.29	1.6	1.8 4X	11.48	2.1	4.0 4X	10.87	3.3
1.0 5X	9.15	1.8	1.4 5X	14.16	2.6	1.8 5X	10.77	2.0	4.0 5X	11.42	3.7
$\bar{x}$	11.53	2.0	$\bar{x}$	11.21	1.9	$\bar{x}$	10.54	2.1	$\bar{x}$	10.64	3.4
SD	1.44	0.3	SD	2.06	0.4	SD	0.96	0.1	SD	0.36	0.3
1.0 1Y	11.84	2.2	1.4 1Y	15.82	2.8	1.8 1Y	14.85	3.4	4.0 1Y	10.36	3.2
1.0 2Y	11.65	2.3	1.4 2Y	16.04	2.4	1.8 2Y	14.97	3.3	4.0 2Y	10.32	3.5
1.0 3Y	12.37	2.2	1.4 3Y	16.37	2.6	1.8 3Y	14.83	3.4	4.0 3Y	10.55	3.4
1.0 4Y	12.17	3.0	1.4 4Y	16.04	2.5	1.8 4Y	13.40	3.0	4.0 4Y	10.08	3.9
1.0 5Y	10.17	1.8	1.4 5Y	16.87	2.9	1.8 5Y	14.59	3.8	4.0 5Y	10.47	3.5
$\bar{x}$	11.64	2.3	$\bar{x}$	16.23	2.6	$\bar{x}$	14.53	3.4	$\bar{x}$	10.35	3.5
SD	0.78	0.4	SD	0.36	0.2	SD	0.58	0.2	SD	0.16	0.2
1.0 Z	-	-	1.4 Z	-	-	1.8 Z	-	-	4.0 2Z	0.94	1.8

**Table 4:** Modulus of elasticity (in MPa) obtained for the PLA + bronze specimens.

No.	*E*	No.	*E*	No.	*E*	No.	*E*
1.0 1X	615.87	1.4 1X	611.03	1.8 1X	514.2	4.0 1X	289.22
1.0 2X	699.13	1.4 2X	534.38	1.8 2X	489.62	4.0 2X	382.03
1.0 3X	534.03	1.4 3X	616.37	1.8 3X	510.9	4.0 3X	277.45
1.0 4X	601.02	1.4 4X	713.67	1.8 4X	546.96	4.0 4X	345.38
1.0 5X	535.92	1.4 5X	547.15	1.8 5X	549.68	4.0 5X	320.1
$\bar{x}$	597.19	$\bar{x}$	604.52	$\bar{x}$	522.27	$\bar{x}$	322.84
SD	60.83	SD	63.73	SD	22.90	SD	37.97
1.0 1Y	546.35	1.4 1Y	602.4	1.8 1Y	454.61	4.0 1Y	332.31
1.0 2Y	525.07	1.4 2Y	674.22	1.8 2Y	470.03	4.0 2Y	300.46
1.0 3Y	582.52	1.4 3Y	634.29	1.8 3Y	442.53	4.0 3Y	324.94
1.0 4Y	424.94	1.4 4Y	643.81	1.8 4Y	463.12	4.0 4Y	264.78
1.0 5Y	570.89	1.4 5Y	591.34	1.8 5Y	380.77	4.0 5Y	306.57
$\bar{x}$	529.95	$\bar{x}$	629.21	$\bar{x}$	442.21	$\bar{x}$	305.81
SD	56.14	SD	29.72	SD	32.06	SD	23.58
1.0 Z	-	1.4 Z	-	1.8 Z	-	4.0 2Z	104.24

## Data Availability

Not applicable.

## References

[1] Liu Z., Wang Y., Wu B., Cui C., Guo Y., Yan C. (2019). A Critical Review of Fused Deposition Modeling 3D Printing Technology in Manufacturing Polylactic Acid Parts. Int. J. Adv. Manuf. Technol..

[2] Cantrell J.T., Rohde S., Damiani D., Gurnani R., DiSandro L., Anton J., Young A., Jerez A., Steinbach D., Kroese C. (2017). Experimental Characterization of the Mechanical Properties of 3D-Printed ABS and Polycarbonate Parts. RAPID Prototyp. J..

[3] Serra T., Planell J.A., Navarro M. (2013). High-Resolution PLA-Based Composite Scaffolds via 3-D Printing Technology. Acta Biomater..

[4] Murariu M., Dubois P. (2016). PLA Composites: From Production to Properties. Adv. Drug Deliv. Rev..

[5] Bajpai P.K., Singh I., Madaan J. (2013). Tribological Behavior of Natural Fiber Reinforced PLA Composites. Wear.

[6] Hanon M.M., Marczis R., Zsidai L. (2019). Anisotropy Evaluation of Different Raster Directions, Spatial Orientations, and Fill Percentage of 3D Printed PETG Tensile Test Specimens. Key Engineering Materials.

[7] Allum J., Gleadall A., Silberschmidt V.V. (2020). Fracture of 3D-Printed Polymers: Crucial Role of Filament-Scale Geometric Features. Eng. Fract. Mech..

[8] Wang X., Jiang M., Zhou Z., Gou J., Hui D. (2017). 3D Printing of Polymer Matrix Composites: A Review and Prospective. Compos. Part B-Eng..

[9] Bochnia J., Blasiak M., Kozior T. (2020). Tensile Strength Analysis of Thin-Walled Polymer Glass Fiber Reinforced Manufactured by 3D Printing Technology. Polymers.

[10] Tekinalp H.L., Kunc V., Velez-Garcia G.M., Duty C.E., Love L.J., Naskar A.K., Blue C.A., Ozcan S. (2014). Highly Oriented Carbon Fiber-Polymer Composites via Additive Manufacturing. Compos. Sci. Technol..

[11] Bochnia J., Blasiak M., Kozior T. (2021). A Comparative Study of the Mechanical Properties of FDM 3D Prints Madeof PLA and Carbon Fiber-Reinforced PLA for Thin-Walled Applications. Materials.

[12] Kariz M., Sernek M., Obucina M., Kuzman M.K. (2018). Effect of Wood Content in FDM Filament on Properties of 3D Printed Parts. Mater. Today Commun..

[13] Ertane E.G., Dorner-Reisel A., Baran O., Welzel T., Matner V., Svoboda S. (2018). Processing and Wear Behaviour of 3D Printed PLA Reinforced with Biogenic Carbon. Adv. Tribol..

[14] Hanon M.M., Alshammas Y., Zsidai L. (2020). Effect of Print Orientation and Bronze Existence on Tribological and Mechanical Properties of 3D-Printed Bronze/PLA Composite. Int. J. Adv. Manuf. Technol..

[15] Unlu B.S., Uzkut M., Atik E. (2010). Tribological Behaviors of Polymer-Based Particle-Reinforced PTFE Composite Bearings. J. Reinf. Plast. Compos..

[16] Unal H., Kurtulus E., Mimaroglu A., Aydin M. (2010). Tribological Performance of PTFE Bronze Filled Composites under Wide Range of Application Conditions. J. Reinf. Plast. Compos..

[17] Sava M., Nagy R., Menyhardt K. (2021). Characteristics of 3D Printable Bronze PLA-Based Filament Composites for Gaskets. Materials.

[18] Sneha P., Balamurugan K., Deepak B.B.V.L., Bahubalendruni M.V.A.R., Parhi D.R.K., Biswal B.B. (2023). Investigation on Wear Characteristics of a PLA-14% Bronze Composite Filament. Recent Trends in Product Design and Intelligent Manufacturing Systems.

[19] Sneha P., Balamurugan K., Kalusuraman G. (2020). Effects of Fused Deposition Model Parameters on PLA-Bz Composite Filament. IOP Conf. Ser. Mater. Sci. Eng..

[20] Sneha N., Balamurugan K. Micro-Drilling Optimization Study Using RSM on PLA-Bronze Composite Filament Printed Using FDM. Proceedings of the 2022 IEEE 2nd Mysore Sub Section International Conference (MysuruCon).

[21] Wei X., Behm I., Winkler T., Scharf S., Li X., Baehr R. (2022). Experimental Study on Metal Parts under Variable 3D Printing and Sintering Orientations Using Bronze/PLA Hybrid Filament Coupled with Fused Filament Fabrication. Materials.

[22] Dizon J.R.C., Espera A.H., Chen Q., Advincula R.C. (2018). Mechanical Characterization of 3D-Printed Polymers. Addit. Manuf..

[23] Saharudin M.S., Hajnys J., Kozior T., Gogolewski D., Zmarzły P. (2021). Quality of Surface Texture and Mechanical Properties of PLA and PA-Based Material Reinforced with Carbon Fibers Manufactured by FDM and CFF 3D Printing Technologies. Polymers.

[24] Kozior T., Bochnia J., Gogolewski D., Zmarzły P., Rudnik M., Szot W., Szczygieł P., Musiałek M. (2022). Analysis of Metrological Quality and Mechanical Properties of Models Manufactured with Photo-Curing PolyJet Matrix Technology for Medical Applications. Polymers.

[25] (2012). Plastics-Determination of Tensile Properties.

[26] Forfutura (2016). MetalFil^TM^-Ancient Bronze-Technical Data Sheet.

[27] (2019). Plastics—Methods for Determining the Density of Non-Cellular Plastics—Part 1: Immersion Method, Liquid Pycnometer Method and Titration Method.

[28] (2022). Plastics—Thermoplastic Materials—Determination of Vicat Softening Temperature (VST).

